# Docosahexaenoic Acid Helps to Lessen Extinction Memory in Rats

**DOI:** 10.3390/molecules23020451

**Published:** 2018-02-18

**Authors:** Michio Hashimoto, Shahdat Hossain, Masanori Katakura, Abdullah Al Mamun, Osamu Shido

**Affiliations:** 1 Department of Environmental Physiology, Shimane University Faculty of Medicine, Izumo, Shimane 693-8501, Japan; shahdat@dhaka.net or shahdat@juniv.edu (S.H.); katakura@med.shimane-u.ac.jp (M.K.); mamun104@gmail.com (A.A.M.); o-shido@med.shimane-u.ac.jp (O.S.); 2 Department of Biochemistry & Molecular Biology, Jahangirnagar University, Savar, Dhaka 1342, Bangladesh

**Keywords:** DHA, fear, extinction memory, BDNF, TrKB, GRP, GRPR, NR2A, NR2B, VAChT, PSD-95

## Abstract

Memory extinction is referred to as a learning process in which a conditioned response (CR) progressively reduces over time as an animal learns to uncouple a response from a stimulus. Extinction occurs when the rat is placed into a context without shock after training. Docosahexaenoic acid (DHA, C22:6, n-3) is implicated in memory formation in mammalian brains. In a two-way active shuttle-avoidance apparatus, we examined whether DHA affects the extinction memory and the expression of brain cognition-related proteins, including gastrin-releasing peptide receptor (GRPR), brain-derived neurotrophic factor receptor (BDNFR) tyrosine kinase receptor B (TrKB), and N-methyl-d-aspartate receptor (NMDAR) subunits NR2A and NR2B. Also, the protein levels of GRP, BDNF, postsynaptic density protein-95 (PSD-95), and vesicular acetylcholine transporter (VAChT), and the antioxidative potentials, in terms of lipid peroxide (LPO) and reactive oxygen species (ROS), were examined in the hippocampus. During the acquisition phase, the rats received a conditioned stimulus (CS-tone) paired with an unconditioned stimulus (UCS foot shock) for three consecutive days (Sessions S1, S2, and S3, each consisting of 30-trials) after 12 weeks of oral administration of DHA. After a three-day interval, the rats were re-subjected to two extinction sessions (S4, S5), each comprising 30 trials of CS alone. During the acquisition training in S1, the shock-related avoidance frequency (acquisition memory) was significantly higher in the DHA-administered rats compared with the control rats. The avoidance frequency, however, decreased with successive acquisition trainings in sessions S2 and S3. When the rats were subjected to the extinction sessions after a break for consolidation, the conditioned response (CR) was also significantly higher in the DHA-administered rats. Interestingly, the freezing responses (frequency and time) also significantly decreased in the DHA-administered rats, thus suggesting that a higher coping capacity was present during fear stress in the DHA-administered rats. DHA treatments increased the mRNA levels of GRPR, BDNF receptor TrKB, and NMDAR subunit NR2B. DHA also increased the protein levels of GRP, BDNF, PSD-95, and VAChT, and the antioxidative potentials in the hippocampus. These results suggest the usefulness of DHA for treating stress disorders.

## 1. Introduction

We have previously reported that docosahexaenoic acid (DHA, C22:6 n-3) augments spatial memory acquisition [1], stimulates neurogenesis [2], protects [3] and improves [4] memory impairments of amyloid β peptide_1–40_-infused Alzheimer’s disease (AD) model rats by decreasing amyloidogenesis [5,6], and protects age-related cognitive decline in the elderly [7]. These results and others [8,9,10] demonstrate a robust link between DHA and cognitive health. When fed on a low-n-3 polyunsaturated fatty acid (n-3 PUFAs) diet, rodents may become DHA deficient and subsequently may suffer from anxiety- and depression-like behaviors [11,12] and impairments of memory [13,14]. Moreover, DHA supplements have been shown to attenuate psychiatric stress activity and bipolar disorder [15]. However, the mechanisms through which the molecule DHA affects anxiety, stress, and fear memory remain unclear.

Fear is an adaptive response to provide protection from potential harm in the environment. Nevertheless, when fear is excessive and disproportionate to the situation, it may lead to the development of an anxiety disorder, which, if sustained, causes an impairment of fear extinction. Therefore, fear conditioning and fear extinction animal models can help to examine the neurobiology underlying fear processes and can act as potential biomarkers of anxiety. Although the exact mechanism by which fear stress or related anxiety develops is still unclear, a literature review points to the failure to inhibit or extinguish fear appropriately [16]. Here, extinction refers to the reduction in a conditioned response (CR) (e.g., fear) when a conditioned stimulus (CS) is repeatedly presented in the absence of an unconditioned stimulus (UCS). An extinction deficit of fear memories is implicated in post-traumatic stress disorder (PTSD) [17]. The freezing response is a general reaction to a fearful stress stimulus, commonly observed in animals during traumatic situations. Although we are aware that neurogenesis contributes to cognitive recovery in rats following traumatic brain injury [18], the mechanism is poorly understood. On the basis of positive roles of DHA in neurogenesis [2], DHA may help cognitive recovery and ameliorate fear-associated stress.

We also evaluated the most likely molecular mechanisms of the effects of DHA on fear-related memory and extinction. The gut, one of the largest endocrine organs in the body, secretes numerous peptide hormones primarily in response to nutrient passing through the lumen of the gastrointestinal (GI) tract. Gut hormones have increasingly been implicated in brain functions [19,20]. Interestingly, many of the same gut hormones are also expressed in the central nervous system (CNS), acting to translate metabolic information between the GI tract and the CNS [21,22,23]. The gut hormone gastrin-releasing peptide (GRP) and its receptor gastrin-releasing peptide receptor (GRPR) have been implicated in fear-related responses [24,25,26]. Mice deficient in GRPR show greater fear in associative learning, indicating that receptor agonist could be used for fear-related disorders [27]. In addition, the tyrosine kinase B (TrkB) receptor and its ligand brain-derived neurotrophic factor (BDNF) promote neuronal plasticity in the adult hippocampus [28,29,30]. Synaptic *N*-methyl-d-aspartate receptor (NMDAR) together with the postsynaptic density protein (PSD)-95 [31] are important in the formation of synaptic plasticity and long-term potentiation (LTP) [32]. Furthermore, memory impairment in conditioned fear is also associated with both cholinergic dysfunction [33], involving the vesicular acetylcholine transporter (VAchT) protein, and oxidative stress [1,3,4]. Therefore, we investigated whether DHA affects the molecules affecting fear responses. Finally, our primary aims were to assess the responses of DHA-administered rats to background contextual fear conditioning and whether DHA reduces the conditioned fear response, i.e., facilitates fear extinction.

## 2. Results

### 2.1. Body Weight, Food Intake and Basal Neuromotor Activities

There were no significant differences in body weight and/or food intake between the rat groups (BW: Control group: 371 ± 29 g; DHA group: 380 ± 20 g; Food intake per day: Control group, 22.3 ± 2.5 g; DHA group, 24.1 ± 3.0 g).

During adaptation, the number of door crossings was not statistically significant between the two groups (DHA versus control rats: number of door crossing, 56.1 ± 8.1 versus 63.0 ± 6.5 times/min, *p* = 0.51; percent of total time utilized for door crossings, 12.45 ± 1.8 versus 14.9 ± 2.2%; *p* = 0.40, respectively). Thus, the rats did not differ in locomotor activities.

### 2.2. Acquisition of Avoidance Learning

**Figure 1:**
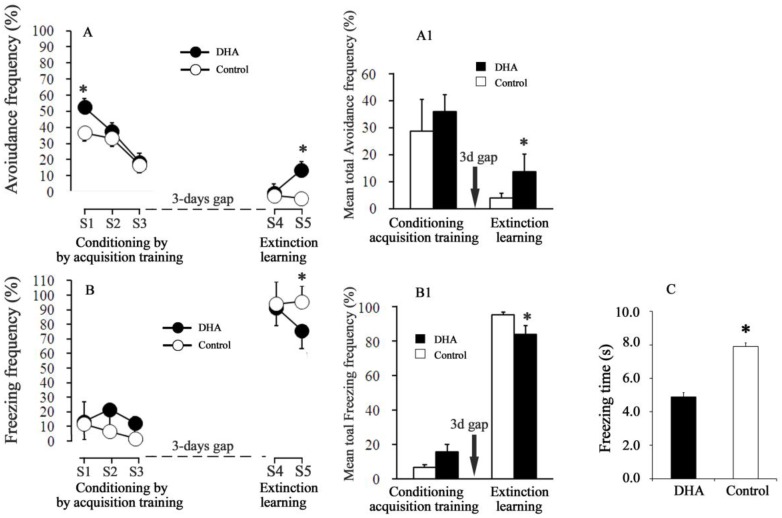
Effects of docosahexaenoic acid (DHA) on fear avoidance (**A**) and freezing responses. (**B)** Freezing frequency in the acquisition trials (S1, S2 and S3) and extinction trials [session4 (S4) and session5 (S5)]. (**C**) Freezing time. S4 and S5 in figure A= Avoidance frequency (%) during extinction S4 and S5, respectively. S4 and S5 in figure B= Freezing frequency (%) during extinction S4 and S5, respectively. Mean total fear-related avoidance and mean total freezing responses are shown in the Figure 1 (**A1**, **B1**), respectively. Black and white circles indicate the results for the DHA and control rats, respectively. The values are represented as mean ± SEM (*n* = 10); * *p* < 0.05 versus control group.

During the acquisition Session 1, the percent of avoidance frequency was significantly higher in the DHA-administered rats (~53%) than in the control rats (~37%) (*p* < 0.001, Figure 1A). In the S2 and S3 acquisition training sessions, the avoidance frequency decreased to 37.5% and 18.3%, respectively, in the DHA-administered rats, while in control rats, the avoidance frequency decreased to 33.3 and 16.6%, respectively. A significant inter-session decrease in fear-related avoidance frequency was evident in the acquisition trials individually for both the DHA and the control groups, as demonstrated by a statistically significant session effect in one-way ANOVA (DHA rats, *F*_(2,21)_ = 35.66, *p* < 0.001; control rats, *F*_(2,21)_ = 11.58, *p* < 0.005; Figure 1A). However, the differences in avoidance frequency between DHA and control rats diminished in the successive acquisition sessions S2 and S3. The escape frequency increased gradually both in the DHA-administered and in control rats during S1→S2→S3 (in DHA-administered rats, 34.1%→40.8%→69.1%; in control rats, 51.7%→60%→81.7%). The freezing frequency (the responses of the rats were not either avoidance or escape response during the whole 30-trial periods) in the S1→S2→S3 sessions were, respectively, 13.3→21.7→12.5% in the DHA-administered rats, whereas in control rats these freezing frequencies in the S1→S2→S3 sessions were, respectively, 11.7%→6.7%→1.7%. The effect of DHA on the freezing frequency was statistically unaffected by DHA treatments during the acquisition trials (S1, S2, and S3) (Figure 1B).

### 2.3. Conditioned Avoidance Response (CR) and Freezing Response (Frequency and Time) during the Extinction Sessions

In the extinction Session S4 (Figure 1A), both the avoidance and escape responses dramatically fell. The conditioned avoidance response (CR) (i.e., the avoidance frequency in the absence of UCS) further reduced in both groups compared with the previous acquisition trial (Session 3). There were no differences in CR between DHA-administered and control rats at S4 (DHA versus control rats at S4: CR = 6.66 versus 5.0%). The escape frequencies (i.e., the movements during the 2nd 5-s of each trial in the absence of UCS) were 0.83 versus 0% in the DHA-administered versus control rats at S4. These results thus suggest that the rats of both groups at S4 did not move to either compartments, rather, they froze infrequently during the whole 30-trials period. In the subsequent extinction session S5, the CR of the DHA-administered rats, however, increased (*p* < 0.05) more than that of the control rats. The ‘degree of fear’ was considered both as percent of freezing frequency and as freezing time per one session. The freezing time during the extinction session S5 was 4.9 ± 0.23 s for the DHA-administered rats, while it was 7.9 ± 0.25 s for the control rats. Fear was quenched to a greater extent in the DHA-administered rats (Figure 1B, Session 5).

#### 2.3.1. Effect of DHA on Hippocampal mRNA Levels of GRPR, TrkB, and NMDAR (NR2A and NR2B Subunits), and on the Protein Levels of GRP, BDNF, PSD-95, and VAchT

**Figure 2:**
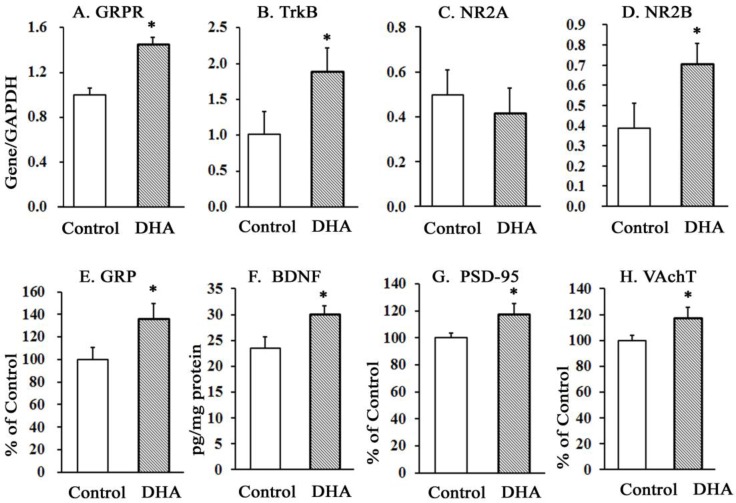
Effect of chronic oral administration of DHA on the mRNA levels (**A**)–(**D**) of hippocampus GRPR, TrkB, NR2A, and NR2B, and on the protein levels (**E**)–(**H**) of GRP, BDNF, PSD-95, and VAchT. The values are mean ± SEM (*n* = 10), each with duplicate determinations; * *p* < 0.05 versus control group, student’s *t*-test.

The oral administration of DHA for 12 weeks significantly increased the mRNA levels of GRPR, TrkB, and NR2B subunits of NMDAR in the hippocampus (Figure 2A,B,D). The levels of NR2A (Figure 2C) were unaffected. The increases in the mRNA levels of GRPR, TrkB, and NR2B receptors were accompanied by increased protein levels of GRP (Figure 2E), BDNF (Figure 2F), PSD-95 (Figure 2G), and VAchT (Figure 2H) in DHA-administered rats compared with control rats.

#### 2.3.2. Effects of DHA Administration on the Oxidative Potentials in Hippocampal Tissues

**Figure 3:**
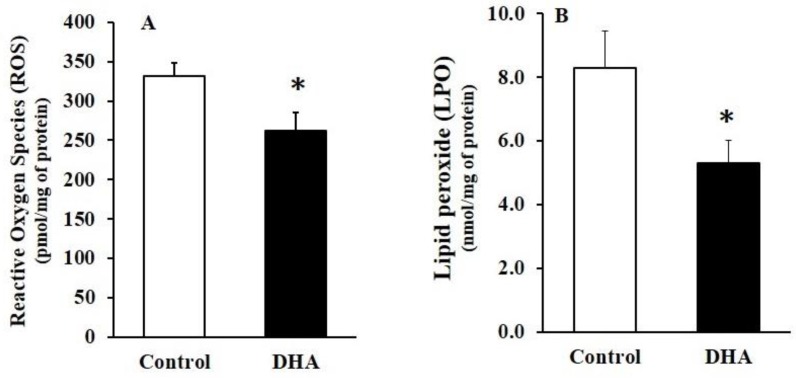
The effect of chronic oral administration of DHA on the levels of reactive oxygen species (ROS) (**A**) and lipid peroxide (LPO) (**B**) in the rat hippocampus. The results are mean ± SEM (*n* = 10), each with duplicate determinations; * *p* < 0.05 versus control group.

The levels of ROS and LPO significantly decreased in the DHA-administered rats compared with the control rats (Figure 3).

#### 2.3.3. Effects of DHA Administration on the Fatty Acid Profiles of the Hippocampus

DHA administration resulted in a significant increase in hippocampal DHA levels, with a concomitant increase in the DHA/arachidonic acid (AA) molar ratio and unsaturation index (USI; Table 1). The hippocampal DHA levels were 45% higher in the DHA-administered rats than in the control rats. The DHA levels also increased in the red blood cell (RBC) membrane and plasma (data not shown) of the DHA rats compared with the control rats. This might indicate that an adequate absorption of dietary DHA occurred in rat blood, and DHA was subsequently delivered to brain tissues, such as the hippocampus.

## 3. Discussion

Compared to the controls, the DHA-administered rats had a higher level of fear-related avoidance memory against shock in the 30-trials acquisition trainings. The rats used in this study were not handled or preconditioned in any specific way prior to the adaptation. Thus, the increased (fear) shock-related acquisition memory in the DHA rats could be attributed to the oral administration of DHA. The data thus suggest that DHA oral administration might have caused “an enhancement of memory acquisition” that enabled the DHA rats to anticipate the conditioned stimulus (CS), i.e., a tone cue as a foreground predictor of shock. The results are thus consistent with our previous reports [1,3,4] that DHA helps in the acquisition of memory. The augmented acquisition of fear memory (in terms of avoidance response >52%) in the DHA rats in Session 1, however, reduced (to 37%) in Session S2 and finally decreased to ~20% at Session S3 (Figure 1). The ability to recognize fear and innate responses by elicitation of stress is essential and critical to survival for all animals. The associative learning processes in Session 1 probably facilitated the prediction of impending threats and allowed the rats to minimize their reaction. This explanation is consistent with the fact that conditioned hypoalgesia develops during fear conditioning to reduce the pain produced by noxious stimuli [34]. Therefore, to more effectively avoid the danger of shock (UCS) during the 30 trials, the rats in the two-way active avoidance shuttle-box exhibited other responses. For example, the escape response, which is like a “get me out of here” reaction aimed at escaping from pain or any sorts of stress. If the floor of the apparatus is electrified (i.e., an UCS is given) and the rat jumps off the shock, the jump is an escape response or behavior. The escape response elicited by an UCS is typically considered a reflexive, unlearned response that is poorly related to cognitive ability. The rats froze during CS; this freezing response is better explained as a new form of learning and is strongly correlated to cognitive brain function. Recently, this response is being referred to as “a time of risk assessment” for the upcoming aversive stimulus. Since the DHA rats exhibited a significantly higher avoidance frequencies compared to the controls during CS–UCS pairing in the acquisition training, one might think that DHA rather seems to enhance the fear avoidance memory. However, during CS-only at the extinction learning, DHA-administered rats exhibited an increased ability to extinguish fear, as indicated by the decreased freezing frequency and time in the DHA-administered rats. These results thus suggest that different forms of memory are involved during acquisition and extinction, and DHA affects both of them. In extinction Session 4, a reduction in the avoidance response occurred in the absence of the UCS in both the DHA-administered and control rats compared with the last conditioning session (during acquisition Session 3; Figure 1). DHA increased (from 6.67% in S4 to 20.8% in S5) the conditioned avoidance responses (CR) in extinction Session 5. In contrast, DHA also reduced (from 92.5% in S4 to 76.76% in S5) the freezing frequency in S5. The freezing time was also reduced significantly in the DHA-administered rats in S5, thus suggesting that DHA yielded a striking, but obviously divergent result in S5. The avoidance response (CAR) in S5 was high, while the freezing frequency was low. Thus, one might think that the results are contradictory. However, we explain this disparity in results in terms of the learning in cognitive processes involved in the avoidance versus the freezing response. Fedorova and Salem (2006) reported that the freezing behavior was dramatically suppressed by supplementation with DHA 48 h after conditioning [35]. Our results are also qualitatively consistent with those of Myers et al. (2006) [36] who reported that extinction initiated at longer delays is thought to employ an inhibitory learning mechanism. Thus, we speculate that DHA might have ameliorated both the inhibitory learning mechanism that underlies the freezing behavior and the excitatory mechanism that underlies the enhanced conditioned (avoidance) response (CAR) in the extinction Session 5. The measures of fear-related avoidance and freezing do not seem by definition to be independent of one another; however, their neurochemical foundation seems quite different, as the ‘avoidance behavior’ employs excitatory mechanisms, while the ‘freezing behavior’ employs inhibitory mechanisms. We hypothesize that these DHA-induced mechanisms are not mutually exclusive because the extinction did not erase the previously paired CS and UCS associations in the DHA-administered rats; again, this is consistent with the notion that extinction and acquisition occur through different mechanisms [37]. In contrast, the control rats exhibited a reduced association (of CS without UCS) capacity in the two-way active avoidance paradigm, i.e., these rats had a lower ability to process the associative change that occurred (absence of UCS) during the extinction sessions, and thus their fear memory remained intact. This is evidenced by the higher percentage of freezing responses (both freezing frequency and time duration) in extinction Session S5 in the control rats. All these justifications thus suggest that DHA can be used in the reduction of fear, i.e., to extinguish fear to some extent in the absence of the original fear-event, i.e., shock.

The impairments in the fear-related conditioned response (CR) and increased freezing time in the control rats coincided with decreases in the protein levels of GRP, BDNF, and PSD-95 and in the mRNA levels of GRP-receptor (GRPR), BDN receptor TrkB, and NR2B subunits of NMDA-receptors (NMDAR) in the hippocampus. Gastrin-releasing peptide (GRP), a bombesin-like peptide, is involved in numerous aspects of brain functions, including conditioned fear responses, memory, and emotional processing [38,39,40,41]. Its binding sites are abundant in the hippocampus [42]. GRP exerts its actions by binding with GRP receptor (GRPR), which is a member of the G-protein coupled receptor superfamily and is widely distributed in the hippocampus, cortex, amygdala, and brain stem [43]. There is considerable interest in dietary n-3 PUFAs, particularly DHA, which has been shown to ameliorate inflammatory bowel diseases in some studies [44]. When DHA reaches the lumen of the GI tract and/or the hippocampus, it is likely to induce GRP–GRPR activity and hence might affect GI tract and hippocampus functions. GRP and GRPR binding might activate PKC or PKA and affect their downstream signaling [45] in neurons.

The GRPR and its ligand GRP are also implicated in memory impairments in patients with AD, transgenic mouse models of AD, and psychiatric disorders [46,47]. Microinfusions of a GRPR antagonist into the hippocampus or amygdala impair the formation of memory for fear conditioning [48], whereas the infusion of a GRPR agonist enhances long-term fear memory consolidation and prevents memory deficits [49]. Thus, GRPR agonistic changes may ameliorate the cognitive impairments. Correspondingly, DHA-induced increases in the levels of GRP and GRPR might have contributed to ameliorate the fear acquisition memory systems of the DHA-administered rats.

NMDARs interact with the BDNF–TrkB pathway to promote synaptic plasticity [50], and BDNF–TrkB signaling is important in the consolidation of fear memories [51] and in emotional regulation [52]. NMDARs remain anchored to PSD-95 that helps in signal trafficking of NMDARs [53] and in LTP regulation [54]. Taken together, the current study indicates that increased levels of these memory-related substrates in the DHA rats may be involved in the improved expression of fear memory acquisition. Memory consolidation and extinction of conditioned fear may be dependent on NMDAR activities in the hippocampus [55,56]. NMDAR consists of 2NR1 and either 2NR2A or 2NR2B [57,58]. The NR2A and NR2B subunits, however, play different roles in the formation of hippocampal memory [59]. While the preferential antagonists to NR2A subunits impair the formation of virtually any type of memory [60,61], hippocampal overexpression of NR2B boosts behavioral performance in several learning and memory tasks [62]. One possibility is that the NR2A and NR2B subunits might act distinctively to regulate downstream signaling. It is however ambiguous which memory processes are regulated by hippocampal NR2B, and how the underlying mechanisms differ from those regulated by NR2A. Intrahippocampal infusion of the NMDAR-agonist d-cycloserine facilitated the extinction and increased NR2B expression [63]. Since hippocampal overexpression of NR2B and/or of NR2B-agonist facilitated the extinction learning, DHA-induced facilitation of fear extinction in the present investigation is compatible with these reports. DHA evidently played a differential role on the expression of NR2A versus NR2B, whereby it tended to suppress NR2A and enhance NR2B. Otherwise, the expression of NR2B could not have increased significantly in the hippocampus of the DHA-administered rats. Our results of increased NR2B levels are qualitatively consistent with the report of Dyall et al. (2007) [64], where a dietary enrichment with n-3 polyunsaturated fatty acids increased the levels of NR2B in the forebrain of aged rats. However, other mechanisms that might have been involved in this process must be delineated to clarify the ultimate effect of DHA on fear conditioning and extinction learning.

In other studies, DHA also increased the hippocampal levels of BDNF [65,66] and TrkB [67]. Our results of increased levels of BDNF and increased mRNA levels of the BDNF receptor TrKB in the hippocampus of the DHA rats are thus qualitatively consistent with these reports [65,66,67]. Therefore, the amelioration of the levels of these proteins might be responsible for the increased acquisition of fear memory.

Again, the mechanism(s) through which DHA contributed to the facilitation of the extinction of the freezing responses appears complex. Neural investigations of extinction demonstrate several similarities to the mechanisms involved in acquisition. For example, GRP reduces freezing and enhances extinction learning [56,68], and the mitogen-activated protein kinase (MAPK) signaling pathway is activated during fear extinction [68,69]. DHA deprivation decreases both MAPK and BDNF levels [70]. Thus, the reversal of MAPK and BDN levels by DHA enrichment is very likely to facilitate fear extinction. Extinction represents inhibitory learning, indicating that it might engage γ-aminobutyric acid (GABAergic) neurons [71]. Impaired GABA_A_ receptor activity is associated with depression, stress, and extinction disorders [72]; GABA_A_–receptor antagonists also enhance the freezing behavior, which can be decreased by DHA supplementation in DHA-deficient rats [73], indicating that DHA might act as a positive modulator of GABA_A_ receptors and improve extinction learning.

Acetylcholine (ACh) is packaged in synaptic vesicles by the vesicular acetylcholine transporter (VAChT). The efficient release of ACh from nerve endings depends on its storage in synaptic vesicles, a step dependent on the activity of a VAChT [74]. The role of ACh and VAChT in learning and extinction memory is still poorly understood, although drugs those enhance cholinergic neurotransmission are used as cognitive enhancers in patients with Alzheimer’s disease [75,76]. Expression of VAChT increased in hippocampus during the acquisition of spatial memory in the Morris water maze [77]. Mice deficient in VAChT exhibited impairments in memory and object discrimination [78]. In a study, de Wilde et al. (2011) [79] reported that Fortasyn™Connect (FC), a multi-nutrient combination containing DHA, increased the count of VAChT-positive cells, conducive to increased VAChT-containing vesicles in the magnocellular basal nucleus. Cholinergic pathways have also been described to act synergistically via NMDA receptors, regulating and leading to synaptic plasticity [79]. The result of increased levels of VAChT and NR2B in the hippocampus concurrently with increases in memory in the DHA-fed rats is thus qualitatively consistent with these reports. However, the exact mechanism remains to be clarified.

A cause–effect relationship between ROS and anxiety is yet to be clarified, although the oxidative imbalance appears to play an important role in fear and anxiety development [80,81]. Anxiety, a feeling of fear, causes the release of stress hormones such as adrenaline, noradrenaline, and cortisol [82]. Thus, we infer that fear stress caused an increase in oxidative stress (OS), which might probably have been induced by the spontaneous release of stress hormones in the plasma and brain tissues, including the hippocampus in our rats. Our inference seems to be consistent, at least partially, with the fact that DHA feeding decreased the levels of ROS in the hippocampus. Numerous other studies have also shown a direct involvement of ROS in anxiety-like behavior in rodents [83,84]. We have also previously reported that DHA-sufficient diet decreased the levels of ROS in the hippocampus and cortical tissues of rat brains [3,4]. Our assumptions are also qualitatively consistent with the reports of Hamazaki et al. (2000) [85], where norepinephrine concentrations decreased significantly, with a concomitant reduction in the degree of anxiety, in DHA-prescribed human subjects. The anti-anxiety and anti-stress effects of DHA were also reported in mice [86,87]. Furthermore, ROS decreasd the levels of BDNF, while DHA supplementation increased it, concurrently with decreases in ROS levels in the hippocampus [65,66]. Other treatments that decrease oxidative stress also corrected the decline in NMDA receptors [88,89], indicating a link between antioxidant levels and the preservation of adequate receptor function. Thus, DHA-induced reductions in ROS levels may positively affect avoidance-related brain cognition.

## 4. Materials and Methods

### 4.1. Animals

Wistar rats (generation zero, G0; *Jcl*; Wistar; Clea Japan. Inc., Tokyo, Japan) were housed, bred, and maintained on a fish oil-deficient diet (F-1^®^; Funabashi Farm, Chiba, Japan) and were allowed ad libitum access to water. The inbred second-generation male rats fed the same F-1 diet were randomly divided into two groups: the DHA group (*n* = 10) and the control group (*n* = 10). The DHA group was orally fed ethyl ester 4, 7, 10, 13, 16, 19-docosahexaenoate (Harima Chemicals, Inc., Tokyo, Japan) emulsified in 5% gum Arabic solution at 300 mg/kg body weight (BW)/day; and the gum Arabic control group was orally fed a similar volume of the 5% gum Arabic solution alone. Oral administration of DHA emulsion or gum Arabic solution was continued initially for 12 weeks. After 12 weeks, the rats were subjected to behavioral experiments, and DHA and Arabic gum feeding were continued up to the end of the experiments. Body weight and food intake were monitored every alternate day. The rats were cared for and killed in accordance with the Guidelines for Animal Experimentation of the Japanese Association for Laboratory Animal Science (Ethics approval number: IZ 27-21).

#### 4.1.1. Shuttle-Box Apparatus

**Figure 4:**
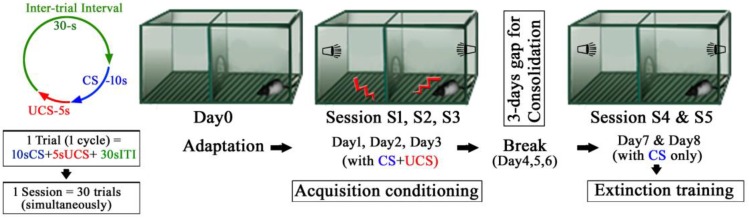
Experimental protocol of the training sessions. Each session consisted of 30 trials with 30 s inter-trial intervals.

The behavior was assessed using the two-way active avoidance paradigm (shuttle avoidance system apparatus; Toyo Sangyo, Toyama, Japan) designed to elicit a conditioned response (CR). The apparatus consisted of two equivalent compartments measuring 56 cm × 21 cm × 25 cm made of clear Plexiglas, with a removable floor made of parallel stainless steel rods that provided a shock grid. A semicircular passage connected the two compartments (Figure 4). Floor shocks were delivered to the grid floor by a ShockStim apparatus unit (San Diego Instruments, San Diego, CA, USA). Auditory tones were presented through a speaker mounted on the chamber wall. The shuttle-box apparatus was placed in a soundproof, dim lighted, windowless room. The experimental protocol is shown in Figure 4. The following sessions, as detailed below, were performed: adaptation, conditioning, extinction.

#### 4.1.2. Adaptation

Each rat was placed into the shuttle-box for adaptation for 450 s. During the adaptation session, no stimuli were presented to the rats. A computerized system recorded rat locomotor activity during the session by infrared photobeams along the sides of each chamber, and the duration of the freezing time was visualized and counted using a video camera.

#### 4.1.3. Conditioning by Acquisition Trials

The rats were placed in the shuttle-box for fear conditioning sessions 24 h after the adaptation session. Fear conditioning was accomplished by pairing the rats subjected to conditioned stimulus (CS) with an unconditioned stimulus (UCS). The CS involved a tone (80 dB white noise, 30 kHz) being delivered for a maximum of 10 s. The UCS involved a foot shock (0.5 mA) delivered to the grid floor for 5 s. An inter trial interval (ITI) varied pseudorandomly between 25 and 30 s, during which the rats were permitted to move freely (Left panel, Figure 4). The avoidance response was defined as the rat travelling from one compartment to the other within 10 s of CS; the UCS and CS were simultaneously terminated with successful avoidance. At the end of the 10 s-CS, the electric foot shock was automatically switched on, the UCS started again and was continued for 5 s until the rat jumped (travelled) to the opposite compartment (this *jumping off* during a UCS is referred to as escape response). If the rats failed to move (escape) to the opposite compartment during the UCS, they received the shock for 5 s. In the subsequent trials, the rats learned to avoid or escape or even tolerate the shocks and exhibited the freezing behavior (response) at or before different time segments of CS (10 s), UCS (5 s), and ITI (30 s) of each trial. The trials were repeated 30 times per session. During conditioning by acquisition trials, each rat underwent a single session for three consecutive days, namely, session 1–3. After each acquisition session, the rats were returned to their home cages.

#### 4.1.4. Extinction Trials

After a three-days break for consolidation, each rat was placed in the shuttle-avoidance apparatus for the fear extinction sessions, which were conducted for two consecutive days by presenting 30 trials of 10 s-CS without 5 s-UCS in session 4 and 5, respectively.

#### 4.1.5. Behavioral Measures

Three parameters were automatically recorded at each session of 30 trials, each consisting of simultaneous (30 cycles) 10 s-CS, 5 s-UCS and 30 s Inter-trial interval, (ITI): (i) avoidance response (number of movements to the opposite safe compartment during CS tone during the first 10 s); (ii) escape response (failed avoidance response after the cessation of the first 10 s of the CS tone, in other words, if a response was made during the second 5 s with UCS); (iii) freezing response (failure of both avoidance and escape responses), when the rats tolerated the shocks and did not move but froze. Since in the extinction sessions the UCS was absent, the avoidance response during the first 10 s was thus a conditioned response (CR), while the escape response was equivalent to movements during the second 5 s. Simply, in extinction trainings, CR = avoidance (movements to the opposite safe compartments) during the first 10 s of CS. Escape = escape (movements to the opposite safe compartments) during the second 5 s, when the shock generator was switched off, i.e., there was no UCS.

All responses were evaluated as percent of the total 30 trials. The percentages of avoidance responses are presented in Figure 1. Because the escape responses were reciprocal to the avoidance responses (i.e., the higher was the avoidance response, the lower the escape response, and vice versa), the data of the escape response were not presented in Figure 1. The freezing frequency was evaluated as the number of ‘no-movements’ other than respiration during each of the 30 trials. The freezing time was defined as the period of time spent in the freezing response during the CS. The freezing time during the extinction trials (during CS only) was considered to be associated with fear extinction. Literally, the greater was the freezing time, the higher the degree of fear was, in other words, a lower freezing time indicated a greater fear extinction.

#### 4.1.6. Preparation of Brain Tissues

After the behavioral studies were completed, the rats were sacrificed. The hippocampus was separated and immediately stored at −80 °C or homogenized in a lysis buffer containing the following: 137 mM NaCl, 20 mM Tris-HCl pH 8.0, 1% NP40, 10% glycerol, 1 mM phenylmethylsulfonyl fluoride, 10 µg/mL protease inhibitor cocktails, 0.1 mM benzethonium chloride, and 0.5 mM sodium vanadate. The homogenates were centrifuged at 13,000× *g* at 4 °C for 30 min, and the supernatants were collected for enzyme-linked immunosorbent assay (ELISA).

#### 4.1.7. Real Time RT-PCR

Total RNA was isolated using an RNA isolation kit (Aurum total RNA Mini kit, Bio-Rad Laboratories, Inc., Hercules, CA, USA), and cDNA was synthesized using the Quantitect reverse transcription kit (Qiagen GmbH, Hilden, Germany) and amplified by the ABI prism 7000 sequence detection system (Applied Biosystems Inc., Foster City, CA, USA). RT-PCR was performed with the Quantitect SYBR green PCR kit (Qiagen, Hilden, Germany). The primer sequences are listed in Table 2. The specificity of the PCR products was confirmed by both melting curve analysis and agarose gel electrophoresis (data not shown). In the initial experiment, we determined the amplification efficiencies of all genes. All amplification efficiencies were comparable (data not shown). The PCR conditions were as follows: initial activation at 95 °C for 15 min, then 40 amplification cycles of denaturation at 94 °C for 15 s, annealing at 58 °C for 30 s, and extension at 72 °C for 30 s. The relative changes in gene expression levels were determined by the 2-ΔCt method described in User Bulletin #2 of the ABI prism 7000 sequence detection system. Glyceraldehyde-3-phosphate dehydrogenase (GAPDH) was used as the housekeeping enzyme.

#### 4.1.8. ELISA

BDNF protein was quantified using a sandwich ELISA kit (BDNF Emax ImmunoAssay System kit, Promega Inc., Fitchburg, WI, USA), according to the manufacturer’s protocol. Indirect ELISA was used for determining the protein levels of GRP, PSDN, and VAchT in the hippocampal samples. In brief, 96-well plates were coated overnight with the hippocampal sample or the respective standard proteins at 4 °C, washed, blocked, and washed again. Then, the plates were incubated with primary antibodies (GRP: rabbit anti-GRP antibody, Abcam; PSD-95: rabbit anit-PSD-95 polyclonal antibody, Invitrogen; goat anti-VAchT polyclonal antibody, Santa Cruz Biotechnologies, Inc., Santa Cruz, CA, USA) and washed again, followed by incubation with the respective horse Radish Peroxidase (HRP)-conjugated secondary antibodies. Finally, the plates were reacted with 100 µL of 3,3′,5,5′-tetramethylbenzidine (TMB) solutions, and the reactions were terminated using 100 μL of 1 M HCl. The absorbance was measured at 450 nm. Hippocampal BDNF, GRP, PSD-95, and VAchT levels were expressed as percentages of the control samples. The titrations of the ELISA assays for BDNF, GRP, PSD-95, and VAchT were performed prior to the final assay. All samples were assessed in triplicate.

#### 4.1.9. Fatty Acid Analyses and Anti-Oxidative Potentials

The fatty acid composition of hippocampal tissue and red blood cell (RBC) membranes was determined by one-step analysis using gas chromatography, and the hippocampal levels of reactive oxygen species (ROS) and lipid peroxide (LPO) were determined as previously described [3,4]. The total protein concentration was determined by the BCA protein assay kit (Pierce, Rockford, IL, USA), with bovine serum albumin as the standard.

#### 4.1.10. Statistical Analysis

Data are presented as mean ± standard error of the mean (SEM). The behavioral data were analyzed by the Kruskal–Wallis analysis of variance (ANOVA), followed by the Mann–Whitney U-test, which was two-tailed when necessary. The comparisons within the same group were performed using the Wilcoxon test. Nonparametric tests were used for data analysis of memory tasks because of the imposition of a ceiling to the performance during behavior. All other data were analyzed using a one-way ANOVA, followed by either the Tukey post hoc test or Student’s t-test for independent samples. The statistical programs used were GB-STATTM 6.5.4 (Dynamic Microsystems, Inc., Silver Spring, MD, USA) and STATVIEW-4.01 (MindVision Software, Abacus Concepts, Inc., Berkeley, CA, USA). A level of *p* < 0.05 was considered statistically significant.

## 5. Conclusions

Relative to controls, DHA caused an improvement in the protein levels of GRP, BDNF, PSD-95, and VAChT in hippocampal tissues. The mRNA levels of GRP receptor (GRPR), BDNF receptor TrKB, NR2B subunit of NMDA receptor also increased in the hippocampus of DHA-administered rats. The amelioration of these parameters could have contributed to the increase in the avoidance and extinction learnings in DHA rats. However, further research is essential to find out the exact mechanism(s) of action of DHA in these types of learning.

## Figures and Tables

**Table 1:** Effect of oral administration of DHA on the fatty acid profile (mol %) of the hippocampus and red blood cell (RBC) membrane.

	Control Group	DHA Group
***Hippocampus***		
AA	6.30 ± 0.35	7.00 ± 0.40
DHA	3.70 ± 0.40	5.40 ± 0.36 *
DHA/AA	0.60 ± 0.04	0.80 ± 0.02 *
USI	61.0 ± 4.0	78.0 ± 3.80 *
***RBC membrane***		
AA	9.40 ± 1.30	10.0 ± 0.70
DHA	1.50 ± 0.13	2.45 ± 0.30 *
DHA/AA	0.20 ± 0.05	0.30 ± 0.02 *
USI	89.0 ± 5.20	100 ± 5.15 *

The values are mean ± SEM; * *p* < 0.05 versus control group. AA, arachidonic acid; DHA, docosahexaenoic acid; DHA/AA, molar ratio of DHA and AA; USI, Unsaturation index. RBC, red blood cell.

**Table 2:** Primer’s list.

Name of the Proteins	Direction of Sequence	Sequence
GRPR	Forward	(5′-GCTGACAGGTACAAAGCCATC-3′)
	Reverse	(5′-GGGTAGGGGGCACAACTAAT-3′)
TrkB	Forward	(5′-GTTGCTGACCAAACCAATCG-3′)
	Reverse	(5′-CATGTACTCAAAGACCATGA-3′)
NR2A	Forward	(5′-CAGCAGCAAGCCACACAGTTATG-3′)
	Reverse	(5′-CAGCAGCAAGCCACACAGTTATG-3′)
NR2B	Forward	(5′-GGACATATCCATGACCAGAAAGAAA-3′)
	Reverse	(5′-GCAACAAACCACAACATTATCGAG-3′)
GADPH	Forward	(5′-ATCTTCTTGTGCAGTGCCAGC-3′)
	Reverse	(5′-CCTTGACTGTGCCGTTGAACT-3′)

GRPR, Gastrin-releasing peptide receptor; TrKB, tyrosine kinase B receptor; NR2, *N*-methyl-d-aspartate receptor subunit 2; GAPDH, glyceraldehyde-3-phosphate dehydrogenase.
